# Development of a primary human Small Intestine-on-a-Chip using biopsy-derived organoids

**DOI:** 10.1038/s41598-018-21201-7

**Published:** 2018-02-13

**Authors:** Magdalena Kasendra, Alessio Tovaglieri, Alexandra Sontheimer-Phelps, Sasan Jalili-Firoozinezhad, Amir Bein, Angeliki Chalkiadaki, William Scholl, Cheng Zhang, Hannah Rickner, Camilla A. Richmond, Hu Li, David T. Breault, Donald E. Ingber

**Affiliations:** 1 Wyss Institute for Biologically Inspired Engineering at Harvard University, Boston, MA 02115 USA; 2 Graduate program, Department of Health Sciences and Technology, ETH Zurich, Zurich, Switzerland; 3 Graduate program, Faculty of Biology, University of Freiburg, Freiburg, Germany; 4 Graduate program, Department of Bioengineering and iBB - Institute for Bioengineering and Biosciences, Instituto Superior Técnico, Universidade de Lisboa, Lisboa, Portugal; 5 Department of Molecular Pharmacology and Experimental Therapeutics, Mayo Clinic College of Medicine, Rochester, MN 55905 USA; 6 Division of Endocrinology, Boston Children’s Hospital, Boston, MA 02115 USA; 7 Division of Gastroenterology, Boston Children’s Hospital, Boston, MA 02115 USA; 8 Department of Pediatrics, Harvard Medical School, Boston, MA 02115 USA; 9 Harvard Stem Cell Institute, Harvard University, Boston, MA 02139 USA; 10 Harvard John A. Paulson School of Engineering and Applied Sciences, Harvard University, Cambridge 02139, MA USA; 11 Vascular Biology Program and Department Surgery, Boston Children’s Hospital and Harvard Medical School, Boston 02115, MA USA; 12 Present Address: Emulate Inc., 27 Drydock Avenue, Boston, MA 02210 USA

## Abstract

Here we describe a method for fabricating a primary human Small Intestine-on-a-Chip (Intestine Chip) containing epithelial cells isolated from healthy regions of intestinal biopsies. The primary epithelial cells are expanded as 3D organoids, dissociated, and cultured on a porous membrane within a microfluidic device with human intestinal microvascular endothelium cultured in a parallel microchannel under flow and cyclic deformation. In the Intestine Chip, the epithelium forms villi-like projections lined by polarized epithelial cells that undergo multi-lineage differentiation similar to that of intestinal organoids, however, these cells expose their apical surfaces to an open lumen and interface with endothelium. Transcriptomic analysis also indicates that the Intestine Chip more closely mimics whole human duodenum *in vivo* when compared to the duodenal organoids used to create the chips. Because fluids flowing through the lumen of the Intestine Chip can be collected continuously, sequential analysis of fluid samples can be used to quantify nutrient digestion, mucus secretion and establishment of intestinal barrier function over a period of multiple days *in vitro*. The Intestine Chip therefore may be useful as a research tool for applications where normal intestinal function is crucial, including studies of metabolism, nutrition, infection, and drug pharmacokinetics, as well as personalized medicine.

## Introduction

The small intestine is the major site for digestion, drug and nutrient absorption, interaction with commensal microbiome, and development of mucosal immunity, as well as a primary site for many diseases, such as bacterial, viral and parasitic infections and inflammatory bowel disease. Because the lack of human-relevant responses has rendered many animal models unsuitable to study causal factors and treatment strategies for human intestinal infections and disorders^1^, three-dimensional (3D) human tissue surrogates, such as intestinal organoids (also known as enteroids) have emerged as promising alternatives. These spheroidal *ex vivo* tissue cultures include Lgr5^+^ intestinal stem cells^2^ and are grown embedded within a complex extracellular matrix (ECM) gel (Matrigel) with Wnt3a, epidermal growth factor (EGF), Noggin and R-spondin 1 (collectively, WENR) to support their indefinite propagation^3,4^. Organoids faithfully recapitulate the cellular diversity of the intestinal epithelium and are ideally suited for *in situ* visualization and continuous monitoring of epithelial development and differentiation^4–8^. However, the presence of an enclosed lumen is non-physiological, as secreted material from goblet, enteroendocrine and Paneth cells, as well as shed apoptotic cells, accumulate within this central space instead of being removed through peristalsis and luminal flow. In addition, the inaccessibility of apical cell surface renders the use of organoids experimentally challenging for transport studies as well as exposure to living commensal microbiome or pathogenic bacteria for more than approximately one day in culture. Finally, organoid cultures lack a tissue-tissue interface, mechanical forces (fluid flow and peristalsis-like motions), immune cells, and a vascular compartment, which are all key contributors to normal intestinal physiology and disease development. Thus, there still remains a compelling need for more complex and physiologically relevant intestinal organ culture systems.

One alternative approach involves the use of 2-channel Organs-on-Chips (Organ Chips), which are microfluidic cell culture devices that contain two parallel hollow culture chambers lined by living human cells and separated by a porous ECM-coated membrane. These chips recapitulate normal tissue-tissue interfaces and mimic the complex physical and biochemical microenvironment of living human organs^9–23^. This technology has been previously applied to develop human Gut Chips that emulate many features of human intestinal structure and function, however, these studies utilized established human intestinal cell lines, such as Caco-2 or HT-29 cells^19–21,24^, which were originally isolated from tumor samples and they harbor multiple gene mutations. In these studies, the intestinal cells also were either cultured alone or in the presence of a non-specialized endothelium (e.g., human umbilical vein endothelial cells)^21^. Thus, these human Gut Chips may not fully recapitulate normal human intestinal functions, and they would be inappropriate to use to study human conditions where genome fidelity is important (e.g., intestinal cancer, drug development, etc.). Other investigators have engineered *in vitro* intestine models using fetal intestinal tissue explants, but these progressively deteriorate after 24 h of culture^25,26^. Thus, in the present study, we set out to develop a primary human Small Intestine Chip (Intestine Chip) using an approach that combines two of the most advanced tissue engineering technologies: intestinal organoids^3,4,27^ and Organ Chips^9,10^.

The Intestine Chip contains normal human intestinal epithelial cells derived from organoids established from endoscopic biopsies or tissue resections of living human intestine, and intestinal tissue-specific microvascular endothelial cells. This microengineered environment recapitulates many key anatomical and functional features of its *in vivo* small intestine counterpart including 3D intestinal villi-like structures, multi-lineage differentiation, epithelial barrier function, enzymatic activity of brush border enzyme and mucus production. Importantly, the transcriptome of the primary Intestine Chip more closely resembles that of adult human duodenum *in vivo* than the organoids that were used to plate the chips or other currently available intestinal cell culture models, including our previous Caco-2 Gut Chip, especially with regard to expression of genes relating to digestion, response to nutrients, cell proliferation, and host defense response to infection.

## Results

### Primary human Intestine Chip developed using biopsy-derived organoids

**Figure 1:**
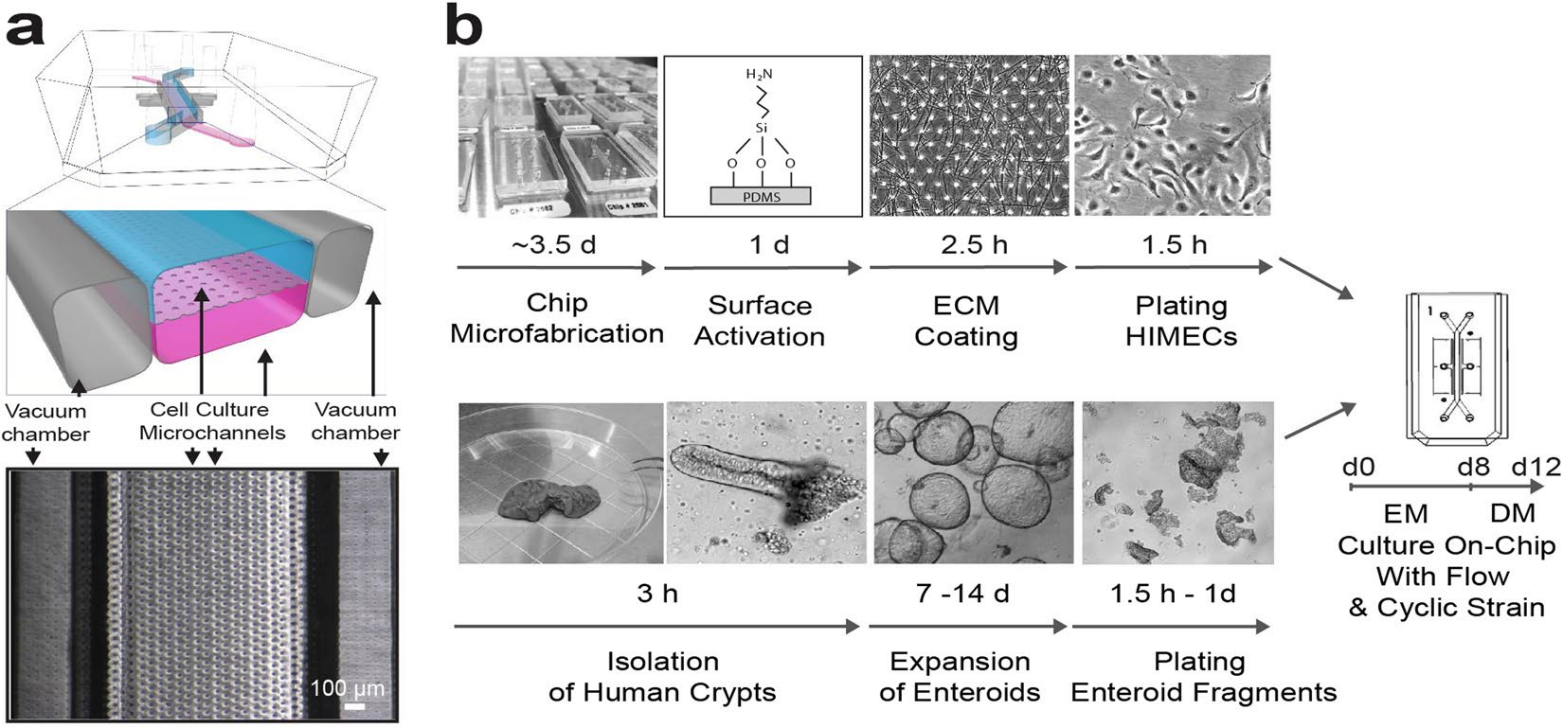
Fabrication of the primary human Intestine Chip. (**a**) A schematic cross-sectional view (top) and a phase contrast micrograph of the chip viewed from above (bottom) showing the upper (epithelial; blue) and lower (microvascular; pink) cell culture microchannels separated by a porous, ECM-coated, PDMS membrane sandwiched in-between. The membrane is elastic and can be extended and retracted by the application of cyclic vacuum to the hollow side chambers. This actuation causes outward deflection of the vertical side walls and lateral extension of the attached horizontal, porous elastic membrane, which induces mechanical deformation of the adherent tissue layers cultured in the central channels. (**b**) Schematic representation of the step-by-step procedure involved in the establishment of microfluidic co-cultures of primary human intestinal epithelium and intestinal microvascular endothelium in the Intestine Chip.

We set out to create an Organ Chip-based surrogate of the human small intestine that incorporates biopsy-derived epithelium, intestinal endothelial cells, physiological fluid flow and peristalsis-like mechanical motions that would allow analysis of human intestinal physiology and pathophysiology in a more *in vivo*-relevant culture microenvironment. To accomplish this, we first established organoid cultures using intestinal crypts derived from macroscopically normal regions of human intestinal endoscopic biopsies. Once the organoids matured (after 5 to 25 passages in culture), we then released organoid fragments through enzymatic treatment and finally seeded these fragments on the upper surface of the ECM-coated porous membrane of a microfluidic Organ Chip (Fig. 1). The polydimethylsiloxane (PDMS) chip devices we used contain two parallel, cell culture microchannels: an upper ‘epithelial’ channel (1 mm high × 1 mm wide) and a lower ‘vascular’ channel (0.2 mm high x 1 mm wide) separated by a thin (50 μm) flexible PDMS membrane containing multiple pores (7 μm diameter, 40 μm spacing) coated with ECM (type I collagen and Matrigel) (Fig. 1a). Each microchannel has a dedicated inlet and outlet for the inoculation of human cells, molecules or microbes as well as for the precise control of physicochemical parameters through the perfusion of laminar flow of appropriate culture medium. Dedicated outlets provide means to collect effluents from the individual chambers for downstream characterization. The upper epithelial channel and lower vascular channel are surrounded on either side by two hollow (1 mm high × 300 µm wide) chambers that permit application of cyclic suction to mechanically stretch and relax the sidewalls, as well as the attached flexible PDMS membrane and adherent tissues in the central channel, thereby emulating peristaltic motions of a living human small intestine.

To obtain primary human intestinal epithelial cells, we utilized a previously published cell culture model based on 3D propagation of human intestinal crypts containing functional stem cells to create a bank of organoids derived from normal duodenal endoscopic biopsies or surgical specimens^3,4,28^. Duodenal organoids cultured for 3–5 days after passaging in expansion medium (EM) were dissociated into fragments and plated in the epithelial channel of the microfluidic devices (Fig. 1b). Organoids from the duodenal region of the small intestine were used in this study because they display higher culture efficiency than those formed from ileum or jejunum, as previously described^28^.

**Figure 2:**
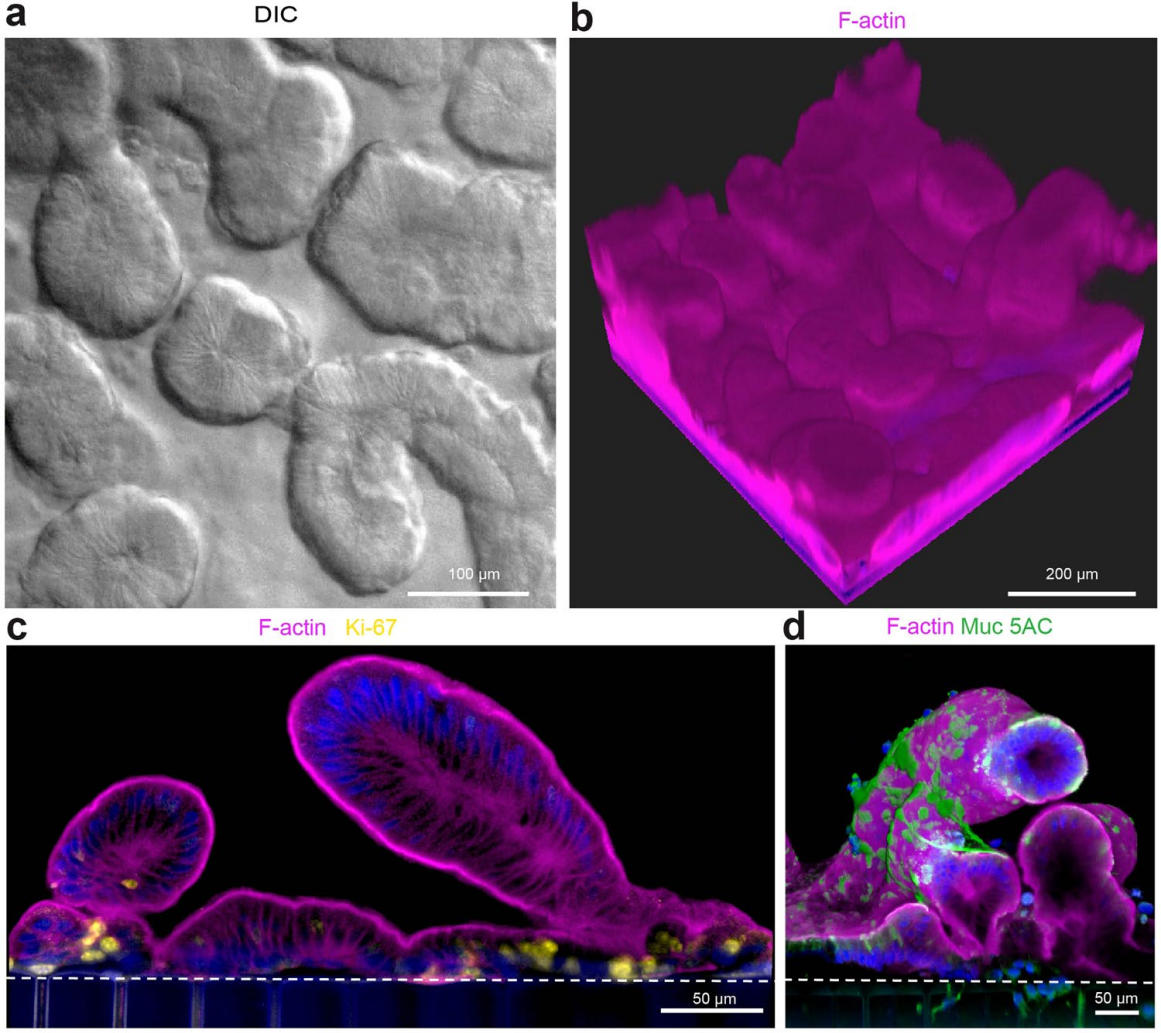
Morphological analysis of Organ Chips lined by primary duodenal organoid-derived epithelial cells in the absence of endothelial cells. (**a**) Microscopic views showing the finger-like potrusions of the primary intestinal epithelium cultured on-chip for 12 days under continuous flow (60 µl hr^−1^), when viewed from above by DIC imaging. (**b**) Representative 3D reconstruction of confocal immunofluorescence micrographs of organoid-derived intestinal epithelium grown on-chip (magenta, F-actin; blue, DAPI-stained nuclei). (**c**) Representative vertical cross sectional view of confocal microscopic images showing intestinal epithelium immunostained for F-actin (magenta) and Ki67 (yellow). (**d**) Representative vertical cross sectional, confocal, micrographic views through the intestinal epithelium-membrane interface of the intestinal epithelium grown on-chip when immunostained for F-actin (magenta) and Muc5AC (green), and nuclei with DAPI (blue) (in c and d, white dashed lines indicate upper surfaces of the porous matrix-coated membrane).

**Figure 3:**
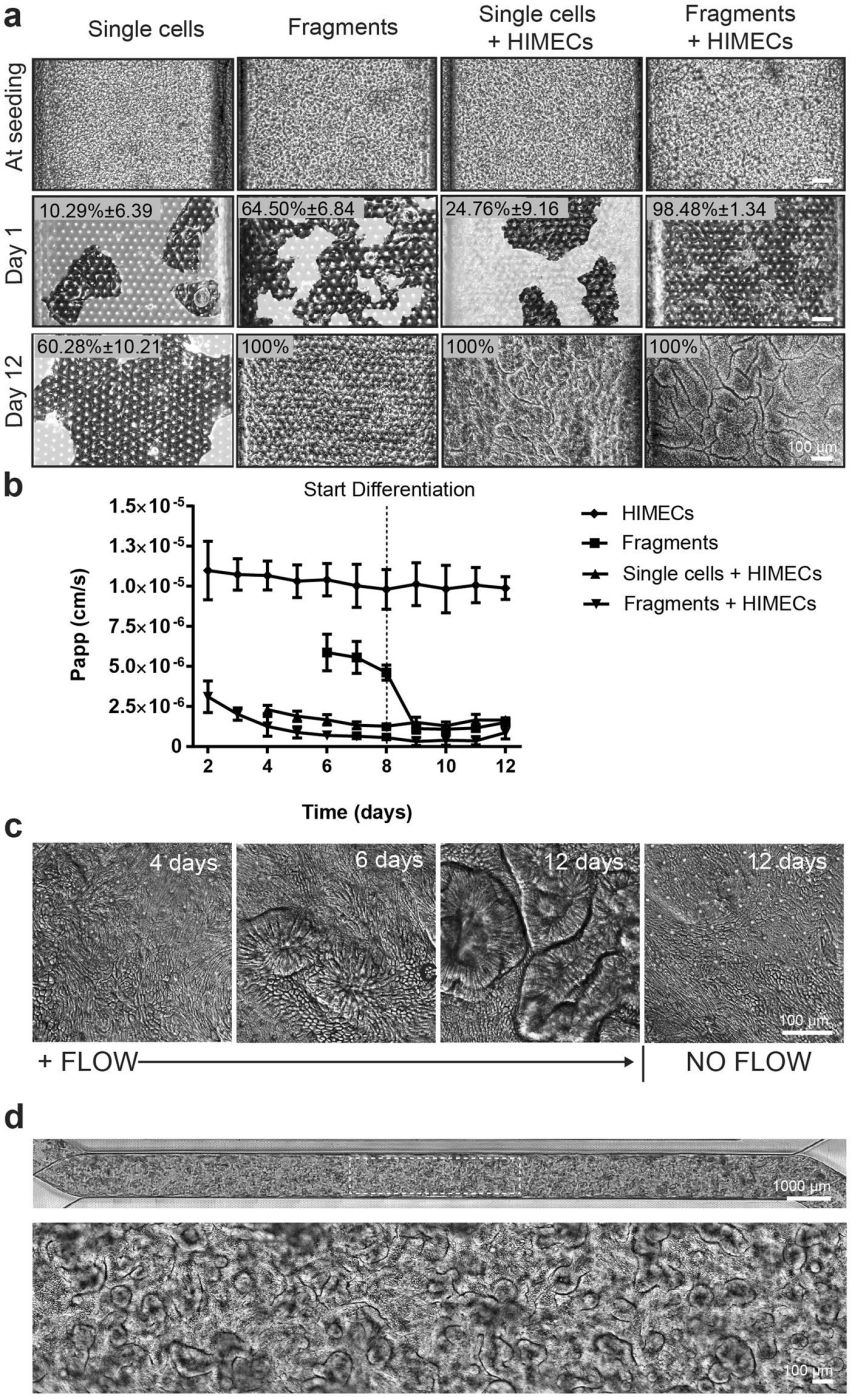
Establishment of the primary human intestinal epithelium in the Organ Chip in the presence or absence intestine-specific microvascular endothelium. (**a**) Comparison of the efficiency of intestinal epithelial cell monolayer formation and development of villi-like structures between Organ Chip devices seeded with single intestinal epithelial cells or organoid fragments in the presence or absence of human intestinal microvascular endothelial cells (HIMECs). All chips were maintained for 1 or 12 days under continuous apical fluid flow of expansion (day 1–8) and differentiation media (day 9–12) and basal perfusion of EGM2-MV medium. White areas delineate empty spaces where initially plated cells detached from the membrane when flow was initiated; dark areas represent patches of attached cells. Graph shows the percentage of the area covered by epithelium at days 1 and 12 of culture, as assessed across 3 different field of views/chip with at least 3 chip replicates per condition, and expressed as mean ±SEM from 3 independent experiments. Note that when EGM2-MV medium was perfused basally, epithelium formed intestinal villi-like structures only when co-cultured with HIMECs seeded on the opposite side of the porous membrane. (**b**) Graph showing maturation of the intestinal barrier function on chips cultured under the same conditions measured by quantifying permeability of fluorescent Lucifer Yellow. P_app_ values are presented as mean ± SEM from 3 independent experiments; only P_app_ values for the cultures that reached 100% of confluency are plotted here. Note that the epithelium develops a higher barrier resistance to Lucifer Yellow more quickly when HIMECs are present. (**c**) Representative differential interference contrast images of the primary intestinal epithelium cultured on chip with HIMECs for 4, 6 or 12 days under continuous flow compared to 12 days in the absence of flow (note that formation of intestinal vill-like structures occurred only in the presence of flow). (**d**) Phase contrast microscopy views of the entire length of the epithelial microchannel (top) and a higher magnification of the area highlighted in the white rectangle (bottom) showing the human primary intestinal epithelium co-cultured with intestinal microvascular endothelial cells in the Organ Chip under peristalsis-like motions and flow for 12 days (culture medium was switched from EM to DM on day 8). Note the presence of villi-like structures across the entire length of the channel.

We first cultured the organoid fragment-derived intestinal epithelial cells alone (without endothelium) in the upper channel, while perfusing both channels with epithelial expansion medium (EM). The large majority of cells initially appeared spread individually across the surface of the porous membrane at the start of the experiment and progressively grew to form a continuous epithelium that underwent villus morphogenesis over 8–12 days (Supplementary Fig. S1a). This resulted in formation of a well-developed intestinal epithelium with a high density of finger-shaped, villus-like protrusions (~30/cm^2^) along the entire length of the upper channel by 12 days of culture, as detected by phase contrast (Supplementary Fig. S1b), differential interference contrast (DIC) (Fig. 2a), and 3D confocal microscopy (Fig. 2b). Immunofluorescence staining of the intestinal epithelium for Ki67 and mucin 5AC (Muc5AC) confirmed the presence of proliferative Ki67-positive cells, which were limited to basal regions of the villi-like structures in close proximity to the PDMS membrane (Fig. 2c), whereas mucin-producing cells were present primarily along the apical regions of the villi-like structures (Fig. 2d), as is observed in living intestine. Computerized image analysis revealed that these villus-like structures were significantly more elongated than the more spherical organoids from which they were derived, with the villi-like structures exhibiting average major and minor axes of 232 ± 17 and 122 ± 7 μm (mean ± SEM) compared to 256 ± 13 and 220 ± 11 μm for the organoids. In the course of these studies, we also found that more effective epithelial cell seeding and efficient monolayer formation can be achieved if organoid fragments are used, as single cell suspensions (produced using longer enzymatic dissociation times) do not expand as efficiently to develop a functional intestinal epithelial barrier (Fig. 3a), and whole organoids remain in their cystic spherical form and thus, never form a continuous monolayer (data not shown).

To create an organ-level model of the human intestine containing a tissue-tissue interface, fragments released from intestinal organoids were seeded on the top of the ECM-coated PDMS membrane, while primary human intestinal microvascular endothelial cells (HIMECs) were plated on the bottom surface of the same membrane in the lower ‘microvascular’ channel; control studies were carried out with epithelium alone in the absence of HIMECs. To maintain the viability and growth of the HIMECs, we perfused endothelial cell medium (EGM2-MV) through the bottom channel while EM was flowed through the top. These studies revealed that cultures that included HIMECs in the microvascular channel required less time to form a confluent epithelium in the upper channel. Organoid fragments achieved confluency within 6 days when seeded alone, while in the presence of endothelium they only required 2 days of co-culture (Fig. 3a). The presence of HIMECs also did not compromise barrier function and possibly enhanced it to a slight degree: with or without HIMEC, the P_app_ was sustained at 1–2 × 10^−6^ cm s^−1^ for up to 12 days of culture (with 8 days of perfusion of EM, followed by 4 days of differentiation (DM) through the upper channel, while EGM2-MV was flowed through the lower channel) (Fig. 3b). After that time, we detected cells that were shed and removed by the fluid flow, as expected from the fast turnover of human intestinal epithelium in which mature enterocytes are shed from the gut lining every 3–5 days^29,30^. The presence of HIMECs in the lower channel also appeared increase the efficiency of monolayer formation when organoids were fully dissociated into single cells prior to culturing them in the microchannel, and formation of putative villi-like structures was also observed in some of these cultures (Fig. 3a). However, the reproducibility of seeding protocol was much higher using organoid fragments versus isolated cells (~90% versus 40% success rate), and thus, we used the fragment method in all subsequent experiments.

Once confluent epithelial and endothelial monolayers were formed in the presence of physiological fluid flow (60 µl h^−1^), the Intestine Chip was exposed to peristalsis-like motions (10% strain, 0.2 Hz) generated by applying cyclic suction to the flexible hollow side chambers. Use of these dynamic co-culture conditions, in combination with a shift from use of the EM to a DM in the upper channel on day 8, led to formation of well-defined intestinal folds throughout the entire length of the epithelial channel (Fig. 3a,c,d). Interestingly, culture of these same cells in the absence of flow did not result in changes of epithelial tissue architecture despite continued application of 10% cyclic strain (Fig. 3c), which is consistent with previous studies using Caco-2 intestinal cells in a Gut Chip^19^.

**Figure 4:**
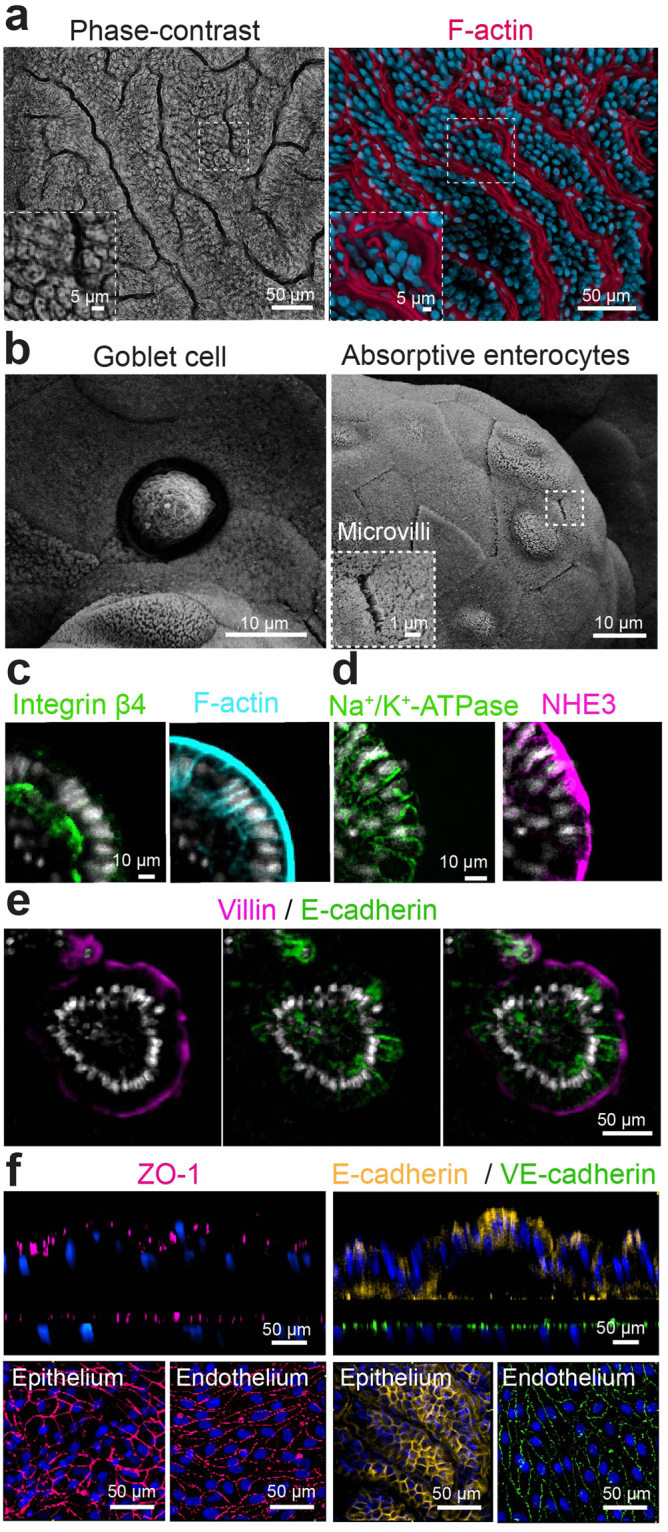
Morphological analysis of the primary human Intestine Chip. (**a**) Representative immunofluorescence microscopic views from above of the intestinal epithelium grown on-chip for 12 days under fluid flow and cyclic strain showing the presence of a continuous brush border along the apical membranes of epithelial villi-like protrusions, which are juxtaposed in close proximity, as visualized by labeling of the brush border for F-actin (magenta) and for nuclei with DAPI (blue). (**b**) High magnification SEMs of the apical surface of the epithelium cultured on-chip showing a goblet cell (left) and absorptive enterocytes (right) with apical microvilli. (**c–e**) Immunofluorescence micrographs of the Intestine Chip showing polarized distribution of apical (F-actin,NHE3, villin) as well as basal (integrin β4) and basolateral (Na^+^/K^+^-ATPase,E-cadherin) proteins in the cross-sectional views of single villus-like structure all counterstained with DAPI (grey).(c) Integrin β4 (green) and F-actin (cyan). (**d**) Na^+^/K^+^-ATPase (green) and NHE3 (magenta), (**e**) Villin (magenta) and E-cadherin (green) (**f**) Confocal immunofluorescence micrographs showing the presence of apical intact tight junctions in the intestinal epithelium and underlying endothelium labeled with ZO-1 (magenta) as well as adherens junctions labeled for E-cadherin (yellow) and VE-cadherin (green); blue indicates DAPI-stained nuclei.

Importantly, phase contrast and confocal fluorescence microscopic analysis of this dynamic tissue-tissue interface confirmed the presence of a continuous, polarized, epithelial cell monolayer with an apical F-actin-containing brush border and basal nuclei aligned along the boundary of each villus-like extension into the lumen of the epithelial microchannel of the chip (Fig. 4a). Scanning electron microscopic (SEM) analysis of the apical surface of the epithelialium lining these villus-like luminal extensions revealed the presence of cells with morphology similar to that previously described in SEM micrographs of mucus-producing goblet cells^31,32^ and well-polarized absorptive enterocytes with densely-packed apical microvilli^33^ (Fig. 4b). Cross-sectional confocal microscopic views of the villi-like structures formed within the primary Intestine Chip confirmed the presence of appropriate epithelial cell polarity with an F-actin-rich brush border at the luminal cell surface and integrin β4 receptors concentrated at the base (Fig. 4c), as well as the major polarized ion transporters, Na^+^/K^+^-ATPase and NHE3 in their normal basolateral and apical positions, respectively (Fig. 4d). NHE3, which is responsible for electroneutral Na^+^ absorption in the small intestine^26^, localized along the apical membranes of the epithelium, while Na^+^/K^+^-ATPase, the major ion transporter responsible for regulating the intracellular Na^+^ gradient necessary for absorption of nutrients^34^, appeared along the basolateral membranes of these cells (Fig. 4d). Reestablishment of normal intestinal epithelial cell polarity was further confirmed by staining for E-cadherin (Fig. 4e,f), which appeared along lateral cell borders, and for villin (Fig. 4e) and zonula occludens-1 (ZO-1) (Fig. 4f) that localized at the cell apex. Thus, in contrast to organoids crossection where the epithelial apex faces an abnormally closed lumen, the horizontal crossectional view of intestinal villi-like stuctures displayed the opposite orientation - with apical cells surface facing upward in contact with fluid flow. Similiarly, immunofluorescence staining of ZO-1 and vascular endothelium (VE)-cadherin demonstrated the presence of well-formed tight and adherens junctions, respectively, in the microvascular endothelium as well (Fig. 4f). Takentogether, these data confirm that we are able to growth primary human intestinal epithelial cells derived from organoids in co-culture with human organ-specific (intestinal) capillary endothelial cells to form a polarized and dynamic intestinal microenvironment using Organ Chip technology.

**Figure 5:**
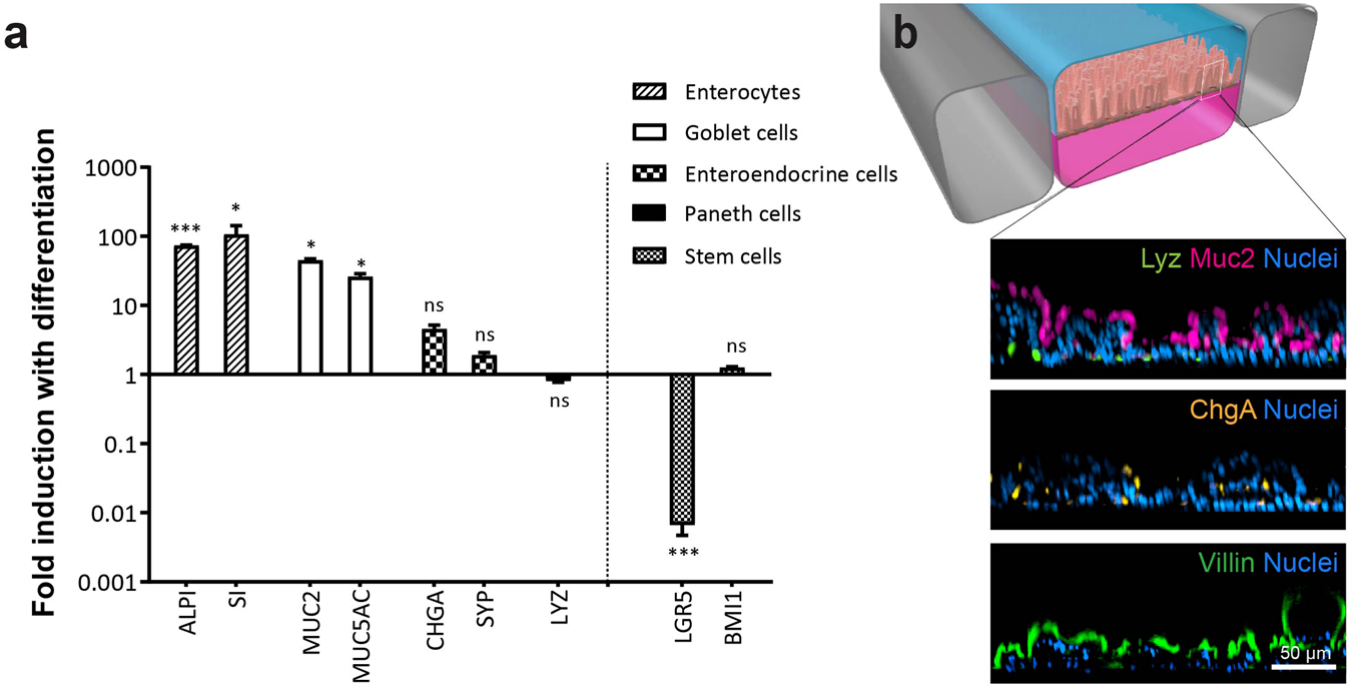
The primary human Intestine Chip exhibits multi-lineage differentiation. (**a**) Graph showing the fold change in the levels of transcripts from epithelial cells cultured in the Intestine Chip and assessed at day 12 relative to the transcript levels in the undifferentiated state assessed at the day 8 (the medium perfused through the apical channel was switched from EM to DM on day 8). Genes analyzed include sucrose-isomaltase (SI) and alkaline phosphatase (ALPI) that are specific for absorptive enterocytes; mucin 2 (MUC2) and mucin 5AC (MUC5AC) for goblet cells; chromogranin A (CHGA) and synaptophysin (SYP) for enteroendocrine cells; lysozyme (LYZ) for Paneth cells; and leucine-rich-repeat-containing G-protein-coupled receptor 5 (LGR5) and polycomb complex protein BMI-1 (BMI1) for stem cells. Values are presented as mean ±SEM from 3 independent experiments involving Intestine Chips generated from organoids isolated from 3 different donors (ns, not significant; *p ≤ 0.05; ***p ≤ 0.001 by Student’s t-test). (**b**) A schematic cross-sectional representation of the 3D intestinal epithelial tissue architecture developed on chip (top) and confocal immunofluorescence micrographs (bottom) showing vertical cross-sections of the differentiated epithelium in Intestine Chip stained for lysozyme (Lyz, green), mucin 2 (Muc2, magenta), chromogranin A (ChgA, yellow) and villin (green). Cell nuclei were counterstained with DAPI (blue).

We next explored whether the primary Intestine Chip faithfully recapitulates normal intestinal differentiation *in vitro*, in which adult intestinal stem cells give the rise to multiple intestinal epithelial cell types, including absorptive enterocytes, enteroendocrine cells, goblet cell and Paneth cells. Removal of Wnt3A and inhibition of Notch signaling, driven by switching the medium in which the epithelial cells are cultured from EM to DM, induced the formation of multiple differentiated intestinal cell lineages. This was demonstrated by detection of increased expression by qRT-PCR of mRNAs encoding alkaline phosphatase and sucrase-isomaltase (absorptive enterocytes); mucin 2 (MUC2) and mucin 5AC (MUC5AC) (goblet cells); and chromogranin A and synaptophysin (SYP) (enteroendocrine cells) (Fig. 5a). Similar results were obtained using primary intestinal epithelial cells isolated from three different donors. Additionally, the increase in intestinal cell differentiation was accompanied by downregulation of expression of the adult intestinal stem cell marker, leucine-rich-repeat-containing G-protein-coupled receptor 5 (LGR5)^35^, whereas there was no detectable change in polycomb complex protein BMI1, which is known to be insensitive to Wnt withdrawal^36^ (Fig. 5a). While expression of the Paneth cell marker, lysozyme, was slightly reduced by Wnt3A removal, we were able to detect the presence of lysozyme-positive cells along with chromogranin A-containing enteroendocrine cells, mucus-producing goblet cells and enterocytes with positive villin-stained apical brush borders in the Intestine Chip by the end of the differentiation process using immunofluorescence microscopy (Fig. 5b), and similar results were obtained in four different Intestine Chips.

### Transcriptomic comparison of the Intestine Chip versus Organoid

**Figure 6:**
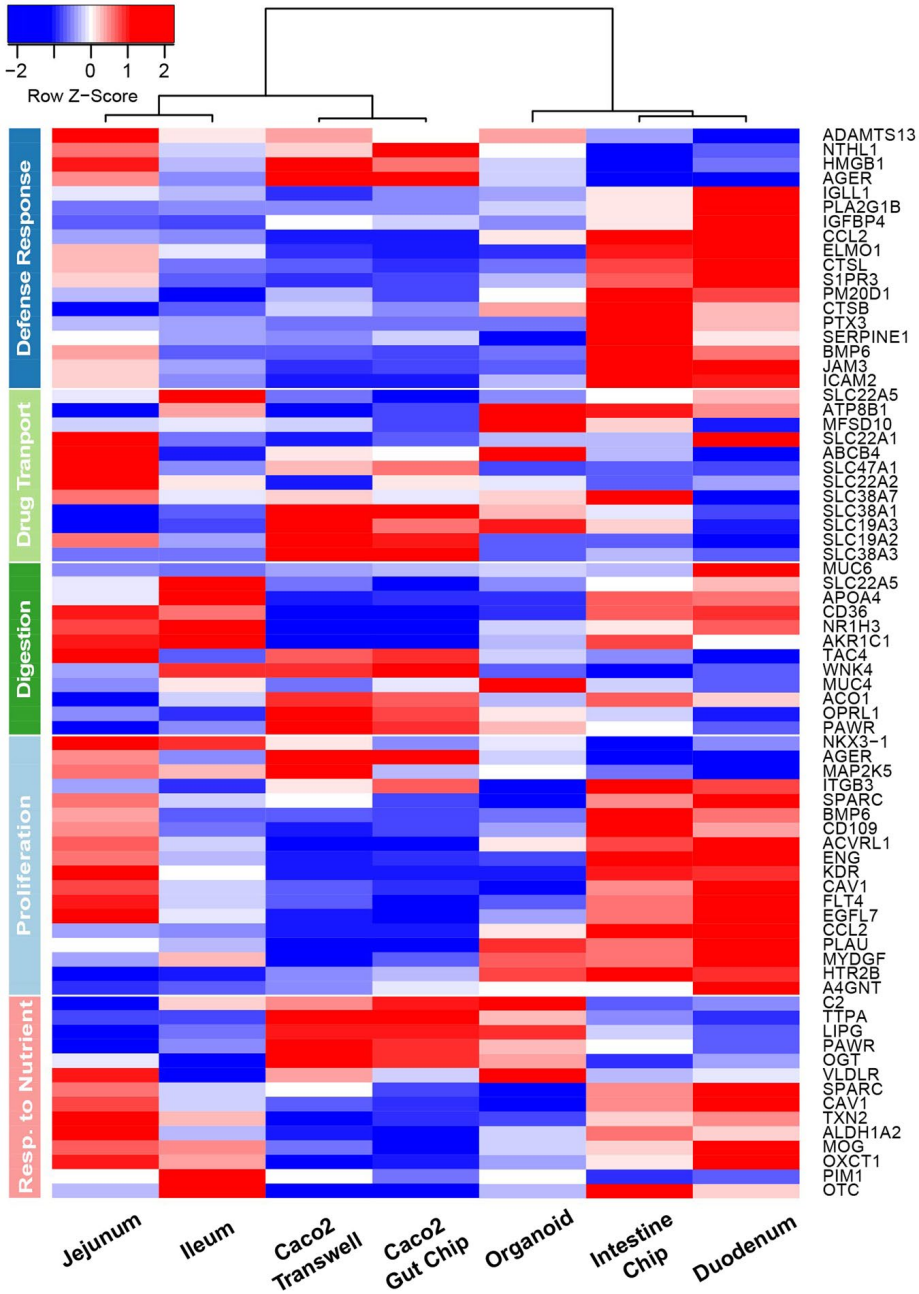
The Intestine Chip resembles human native duodenum. A curated heatmap was generated for genes selected through z-score comparison and template matching method showing genes with similar expression profiles in the mechanically active Intestine Chip (with fluid flow at 60 μL h^−1^ and cyclic stretching at 10%, 0.2 Hz; Intestine Chip) and *in vivo* duodenum in comparison to *in vivo* jejunum and ileum, as well as 3D organoids and Caco-2 based Gut Chip (Caco-2 Gut Chip) and static Caco-2 Transwell models. 3D organoids and Intestine Chip were established from the same duodenal tissue biopsies derived from 3 healthy donors and differentiated for 4 days in DM medium before assessment. Gene expression data for normal human small intestine (duodenum, jejunum, and ileum), Caco-2 Gut Chip, and Caco-2 Transwell were obtained from Gene Expression Omnibus (GEO) database^55,57^. Selected genes shown belong to the following GO terms: Defense Response (GO:0006952), Drug Transport (GO:0015893), Digestive System Process (GO:0022600), Regulation of Epithelial Cell Proliferation (GO:0050678), and Response to Nutrients (GO:0007584). See Supplementary Table S1 for full names of all genes and GO categories.

To further characterize the degree of intestinal differentiation induced on-chip, we carried out transcriptome-wide analysis of the Intestine Chip after 12 days of culture in the presence of flow and peristalsis-like motions, and compared the results with gene profiles obtained from *in vivo* analysis of human ileum, jejunum and duodenum. We also compared these results to those previously obtained with the Caco-2 human Gut Chip and Caco-2 Transwell cultures. Additionally, for the first time, we performed a head-to-head comparison of the Organ Chip approach versus standard intestinal organoid cultures by comparing the transcriptomes of Intestine Chips established from organoids isolated from three different donors and transcriptome profiles of duodenal organoids, which were used for chip seeding. Importantly, both organoids and chips were also cultured under the same conditions: in the presence of EM media for 8 days, followed by 4 days of DM. We initially focused our comparison on genes associated with crucial intestinal functions including digestive function, response to nutrients, drug transport, regulation of intestinal cell proliferation, and host defense response as defined by Gene Ontology (GO) (Fig. 6). As expected, we found that the primary cell-based Intestine Chip and duodenal organoid culture better matched the *in vivo* duodenum transcriptome than the culture models that utilized the Caco-2 cell line, both when we compared these GO gene sets (Fig. 6) and when we analyzed the entire transcriptome (Supplementary Fig. S2). This finding is consistent with past work that showed the human Caco-2 Gut Chip more closely mimics the human ileum^21^. Identification of differentially expressed genes by template matching revealed functional areas, including those related to host defense response to infection, digestion, response to nutrients, drug transport, and cell proliferation where the Intestine Chip is much more similar in phenotype to the living human duodenum than the organoids from which they were derived (Fig. 6). Importantly, comparison of gene profiles across the entire transcriptome by comparing the Pearson’s correlations of Intestine Chip and duodenum (r = 0.6540) versus organoids and duodenum (r = 0.6361) using Fisher’s z transformation confirmed that the Intestine Chip was significantly more similar (z-score = −10.87, *p* = 1.153 × 10^−27^) to the duodenum than the organoid samples. Taken together, these results indicate that the Intestine Chip better recapitulates the morphology, multicellular composition, and gene expression patterns of the intestinal segment from which it was derived than any of the other *in vitro* intestinal culture systems assessed in this study, including 3D intestinal organoids.

### Recapitulation of normal intestinal functions

**Figure 7:**
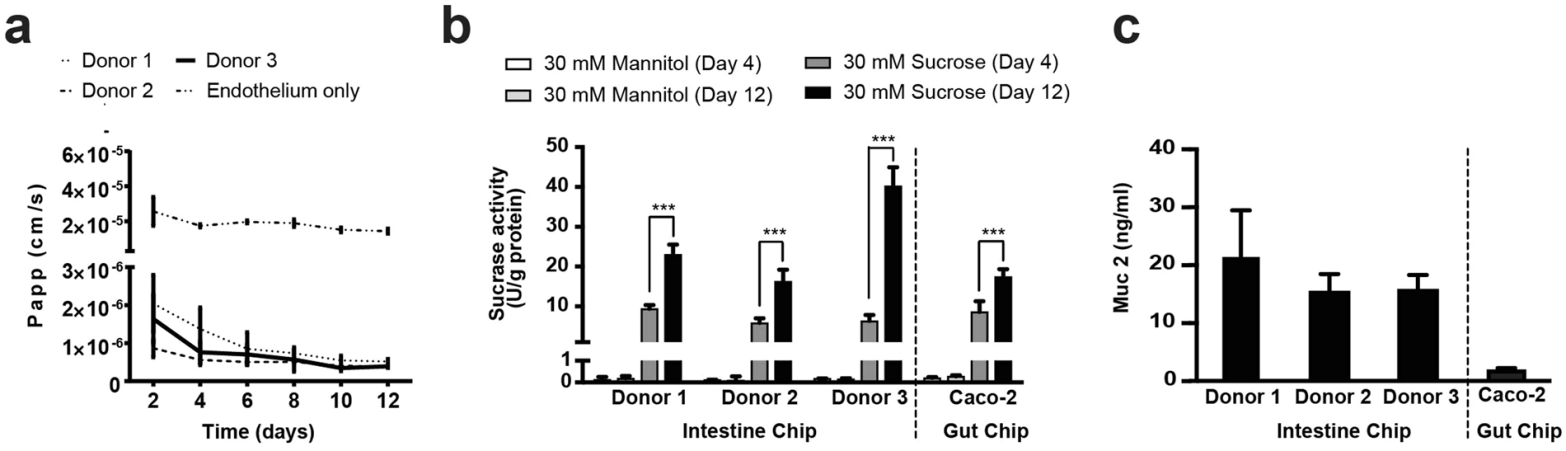
Modeling normal intestinal physiology in the Intestine Chip. (**a**) Comparison of barrier function measured in the intact Intestine Chips lined with epithelium derived from biopsies from 3 different donors and primary HIMECs versus chips lined by endothelium alone by measuring paracellular passage of 450 Da Lucifer Yellow over 2 to 12 days of culture. Data are presented as mean ± SEM of 3 independent experiments involving Intestine Chips generated from organoids derived from three different donors (3 chips/donor). (**b**) Comparison of intestinal epithelial cell sucrase-isomaltase activity in the Intestine Chip and Caco-2 Gut Chip showing that specific enzyme activity increases in both from 4 to 12 days of culture as the cells differentiate into mature absorptive enterocytes. The increased concentration of glucose in the chip effluents results from hydrolysis of 30 mM sucrose which results in formation of glucose and fructose; note that this does not occur when 30 mM mannitol is used instead of sucrose. Data are presented as means ± SEM from 3 independent experiments, each involving 3 different Caco2 Chips and 3 individual chips/organoid donor (***p < 0.001 by ANOVA). (**c**) Comparison of mucin 2 (Muc2) levels in the apical secretions collected in the effluent from the epithelial channel of the Intestine Chip generated from organoids derived from 3 different donors versus that measured in effluents of a human Caco-2 cell line-based Gut Chip at day 12. Data represent means ± SEM from 3 independent experiments involving 3 different Caco-2 Chips or Chips from 3 different donors (3 chips/donor).

To further explore the ability of the primary Intestine Chip to emulate normal intestinal functions in a robust manner, we analyzed the selective permeability of intestinal barrier, the digestive capacity of enterocyte brush border enzymes, and secretion of mucins from goblet cells. Importantly, Intestine Chips from all three donors developed strong intestinal barrier function over a period of 12 days (Fig. 7a) that was similar to what we observed previously (Fig. 3b). These Intestine Chips were then assessed for their digestive capacity by measuring the activity of the brush border enzyme, sucrase-isomaltase, which breaks down sucrose to glucose. Intestine Chips that were infused through the epithelial microchannel either with sucrose, or with the non-metabolizable control disaccharide, mannitol^31^, and enzyme activity was quantified by measuring glucose levels in the apical effluents. These studies revealed similar rates of sucrose hydrolysis in all three differentiated primary Intestine Chips, with glucose production levels that increased over the 12 days of culture and were comparable to those measured in the previously described Caco2 cell-lined Gut Chip (Fig. 7b). These data confirm the presence of functional absorptive enterocytes in the Intestine Chip and suggest that this Organ Chip model of human intestine may be useful for studies of both normal digestion and malabsorption, as well as for analyzing differences in intestinal digestive function between different donors. By extending this model to incorporate organoids isolated from other portions of the human small intestine, we could enhance our ability to analyze nutrient transport and drug absorption (e.g., which occur more frequently in the jejunum), in addition to analyzing regional differences in functional responses (e.g., absorption of lipid and cholesterol occurs primarily in the jejunum, whereas bile acids and vitamin B12 are absorbed in the ileum)^37,38^, which could provide great value when addressing nutrition and pharmacology-related questions in the future.

Another important function of intestinal epithelium is the secretion of mucus, which protects the gut lining from chemical or physical injury as well as enteric pathogens. When we evaluated the presence of MUC2 by ELISA in the apical effluents of the Intestine Chips from the 3 donors, we found that we could detect its presence in all of the chips at a 10 times higher concentration than that measured in the human Gut Chip lined with Caco-2 intestinal cells^19–21^ (Fig. 7c). Thus, the primary Intestine Chip exhibits enhanced functionality relative to the human Caco-2 Gut Chip as well as primary human organoids.

## Discussion

We report here the development of a bioengineered *in vitro* model of the duodenum portion of the human small intestine by combining microfluidic Organ Chip technology with organoid-based methods for culture of primary epithelial stem cells from duodenal biopsies. The Intestine Chip recapitulates important structural features and functions of the native duodenum, including formation of elongated villi-like structures lined by a polarized epithelium expressing markers of enterocytes, goblet cells, enteroendocrine cells and Paneth cells, as well as basal proliferative cells, strong barrier function, brush border digestive enzyme activity, and continuous accessibility to fluids flowing through the apical lumen. This Organ Chip technology differs substantially from past *in vitro* microfluidic models of human intestine, including a microfluidic human Gut Chip^20,21,39^, as it incorporates the use of primary intestinal epithelium isolated from individual patient biopsy-derived organoids as well as gut-specific microvascular endothelial cells; it also support formation of villus-like structures without requiring use of a micropatterned villus-shaped scaffold^40,41^. Importantly, the villi-like projections formed in the Intestine Chip do not exhibit the strict villus/crypt compartmentalization present in native human intestine; however, further improvement could be induced through application of chemical gradients of growth factors (e.g., Wnt-3A, R-spondin and noggin) to the basal-luminal axis or by incorporation of pericryptal mesenchymal cells that secrete factors which are essential for maintenance of intestinal stem cells (e.g., Wnt2b, R-spondin1, and Gremlin1)^40–43^. The mechanism by which these villi-like structures form under flow conditions remains unclear; however, recent findings with the human Gut Chip lined by Caco2 cells suggests that intestinal epithelial cells can establish their own a transepithelial gradient of a Wnt antagonist when cultured under flow, which contributes to this response (Kim *et al*., unpublished data).

The vascularized Organ Chip design we utilized enables application of physiologically relevant mechanical cues, including luminal fluid flow within both the epithelial and vascular channels that is critical for formation of villus-like structures, as well as peristalsis-like cyclic deformations, in addition to providing precise independent control over the chemical composition of culture medium in each channel. The ability to collect effluents of the two separate channels independently also enables dynamic sampling and analysis of biochemical compounds that are produced or secreted by the intestinal epithelial cells or endothelial cells, such as inflammatory cytokines or metabolic products, which is not possible with conventional organoid cultures. Additionally, intestinal epithelial cells grown within Organ Chips could be exposed from the apical side to pathogens, or co-cultured with commensal microbes to model enteric infection or the natural microbiome, as previously demonstrated with the Caco-2 Gut Chip^19^. The presence of the endothelium-lined microchannel (which is absent in organoid cultures) also can enable analysis of absorption of nutrients or oral drug absorption and their bioavailability, as well as characterization of physiologically relevant pharmacokinetic parameters because they are significantly influenced by the passage of drug compounds back and forth across the endothelial-parenchymal tissue-tissue interface. In addition, the presence of the endothelium permits analysis of the contributions of circulating immune cells that may be recruited under physiological flow conditions within microfluidic Organ Chips, as demonstrated in previous studies^11,21,44^. Most importantly, our head-to-head transcriptomic comparison of the Intestine Chip organoids used to supply the cells for the chips revealed that the Intestine Chip more closely mimics living duodenum, and that it better recapitulates many key functions of the living small intestine, including host defense response to infection, cell proliferation, digestion, and response to nutrients, than do the duodenal organoids. Thus, these findings suggest that while organoids may be an outstanding tool to study and probe intestinal stem cell differentiation and histogenesis, they are limited in comparison to the Organ Chip technology when it comes to studying organ-level functions and pathophysiology, particularly in relation to mechanobiology, immunology, infectious disease and drug development.

This primary human Intestine Chip can be adapted for a wide range of applications, including basic research studies on the intestinal development, stem cell maturation and intestinal epithelial cell differentiation; assessment of nutrient transport, sensing, and absorption; intestinal barrier function and tissue-tissue (e.g., epithelial-endothelial) interactions; evaluation of drug delivery; therapeutic efficacy or toxicity; characterization of host-pathogen responses at the mucosal interface; and regenerative medicine studies. In addition, the ability to create Chips from individual donors as demonstrated in this study opens the possibility of creating Intestine Chips lined by cells from individuals with specific genotypic and disease-related phenotypic characteristics. These chips could be used to investigate patient-specific disease mechanisms as well as response to therapies, and thereby help to advance personalized medicine in the future. A recent report suggests that this approach also may be extended to be used with human induced pluripotent stem cell-derived organoids, which could further enhance personalized medicine approaches^45^.

Although future directions include integrating the remaining important components of the living intestine into the Chip, including intestinal fibroblasts, immune cells (e.g., macrophages, intraepithelial lymphocytes and dendritic cells) and the enteric nervous system, previous studies have demonstrated that the power of the Organ Chip technology approach specifically lies in its ability to mimic organ-level complexity by progressively integrating different cell types one-at-a-time and studying the system at varying levels of system complexity^9,10^. Moreover, this organ-level synthetic biology approach permits one to gain insight into mechanisms of biological regulation by manipulating potential contributing physical factors (e.g., flow, peristalsis) and cellular components separately while simultaneously providing a window on molecular-scale biochemical, genetic and cellular responses in real-time. The microengineered nature of this technology also allows users to reconstitute and control molecular gradients (e.g., Wnt, growth factors, etc.), which play a key role in intestinal development and regeneration. Thus, we believe that this primary human Intestine Chip has extraordinary experimental potential for understanding and modeling development, homeostasis and diseases of the human intestine, including intestinal enteropathies, inflammatory bowel disease, celiac disease and cancer.

## Methods

### *In vitro* culture of human intestinal organoids

De-identified endoscopic tissue biopsies were collected from grossly unaffected (macroscopically normal) areas of the duodenum in 10–14 year old patients undergoing endoscopy for gastrointestinal complaints. Informed consent and developmentally-appropriate assent were obtained at Boston Children’s Hospital from the donors’ guardian and the donor, respectively. All methods were carried out in accordance with the Institutional Review Board of Boston Children’s Hospital (Protocol number IRB-P00000529) approval. Tissue was digested in 2 mg ml^−1^ collagenase I for 40 min at 37 °C followed by mechanical dissociation, and isolated crypts were resuspended in growth factor-reduced Matrigel (Becton Dickinson) and polymerized at 37 °C. Organoids were grown in expansion medium (EM) consisting of Advanced DMEM F12 supplemented with L-WRN conditioned medium (50% vol/vol, ATCC, cat. no. CRL-3276)^2^, glutamax, HEPES, murine epidermal growth factor (50 ng ml^−1^), N2 supplement, B27 supplement, 10 nM human [Leu15]-gastrin I, 1 mM n-acetyl cysteine, 10 mM nicotinamide, 10 μM SB202190, 500 nM A83-01, as described^4,6^. Differentiation medium (DM) is EM without L-WRN conditioned medium, nicotinamide and SB202190, but supplemented with 1 μg ml^−1^ human recombinant R-spondin 1 (Peprotech), 100 ng ml^−1^ human recombinant Noggin (Peprotech) and 10 µM γ-secretase inhibitor DAPT, as previously described^3,4,27^. Organoids were passaged every 7 days by incubating in Cell Recovery Solution for 40 min at 4 °C, followed by mechanical dissociation. Organoids were seeded on chips between passage number 5 and 25 and karyotyping was performed to confirm the absence of chromosomal anomalies.

### Intestine Chip culture

Intestine Chips were fabricated from PDMS and assembled as described^19–21^, or they were obtained from Emulate Inc. (Boston, MA). Chips were activated by oxygen plasma treatment for 1.5 min followed by incubation with APTMS (2% vol/vol in ethyl alcohol) for 30 min at RT, washing in ethyl alcohol, and incubating the devices at 80 °C overnight. Type I collagen (200 μg ml^−1^) and Matrigel (1% in PBS) were then introduced into the channels, and incubated in a humidified 37 °C incubator for 2 h before washing with PBS. Epithelial organoids were isolated from Matrigel and the cells dissociated with TrypLE supplemented with 10 μM Y-27632. Epithelial cells were then resuspended in EM (6 × 10^6^ cells/ml; of which 30µl was used to seed each chip resulting in ∼180,000 cells/chip), infused into the top channel, and incubated overnight in static at 37 °C; starting the following day EM was perfused at 60 μl h^−1^ through top and bottom channels until day 8 when the medium was switched to DM for additional 4 days (except for the control experiment shown in Fig. 2 where organoid-derived epithelium was perfused with EM apically and basally throughout the entire course of the experiment).

In studies that included an endothelium, Human Intestinal Microvascular Endothelial Cells (HIMECs; ScienCell) were plated (8 µl of cell suspension at 8 × 10^6^ cells/ml was used to seed each chip resulting in ∼ 64,000 cells/chip) on the lower side of the ECM-coated porous membrane in EGM2-MV medium, which contains human epidermal growth factor, hydrocortisone, vascular endothelial growth factor, human fibroblastic growth factor-B, R3-Insulin-like Growth Factor-1, Ascorbic Acid and 5% fetal bovine serum (Lonza Cat. no. CC-3202). Chips were inverted and incubated under static conditions for up to 1 h at 37 °C to promote HIMEC cell adhesion to the membrane before the epithelial cells were plated. The next day, we initiated continuous flow (60 μl h^−1^) of the EM and EGM2-MV media through the top and bottom channels, respectively. Cyclic, peristalsis-like, membrane deformations (10% strain; 0.2 Hz) were applied after formation of confluent monolayers (3–4 days) using a vacuum pump controlled by an electronic vacuum regulator (ITV009, SMC Corp.) and an Arduino microcontroller.

### Intestinal functional assessment

Lucifer Yellow (450 Da) added to the epithelial channel of the Intestine Chip to assess intestinal barrier permeability. The concentration of dye that diffused through the membrane into endothelial channel was measured in the effluent, and apparent paracellular permeability (*P*_*app*_) was calculated using the following formula:

$${{\boldsymbol{P}}}_{{\boldsymbol{app}}}=\frac{{{\boldsymbol{V}}}_{{\text{rec}}}\cdot {\boldsymbol{d}}{{\boldsymbol{C}}}_{{\boldsymbol{rec}}}}{{\boldsymbol{A}}\cdot {\boldsymbol{dt}}\cdot {{\boldsymbol{C}}}_{{\boldsymbol{don}},{\boldsymbol{t}}=0}}$$

where ***V***_***rec***_ is volume receiver (endothelial compartment), ***C***_***rec***_ concentration receiver, **A** the seeded area and ***C***_***don***_ concentration donor (epithelial compartment).

To measure sucrase activity, upper and lower chamber of the Intestine Chip were perfused PBS with Ca^2^/Mg^2^ for 1 h to remove any residual glucose. 30 mM sucrose reaction buffer was prepared in PBS with Ca^2^/Mg^2^; the NaCl concentration in the PBS was reduced to 120 mM to adjust for osmolarity in the presence of 30 mM sucrose, and the sucrose was replaced with 30 mM mannitol in control samples, as described previously^46^. Sucrose or mannitol reaction buffer was then introduced in the upper microfluidic channel and PBS with Ca^2^/Mg^2^ into the lower channel. Glucose levels were measured using an Amplex Red Glucose assay kit (Thermo Fisher); total protein content in epithelial cell lysates was determined using a Pierce BCA Protein Assay (Thermo Fisher). Glucose concentrations were determined from a standard curve and sucrase activity expressed as units per gram of protein; 1 unit (1 U) = activity that hydrolyzes 1 μmol substrate min^−1^ at 37 °C. To assess MUC2 production, the luminal effluent was collected over night and mucin 2 content was measured using a Human Mucin 2/MUC2 ELISA Kit (LSBio).

### Morphological analysis

Intestine Chips and Organ Chips seeded only with intestinal epithelium were formaldehyde-fixed and permeabilized in 5% (wt/vol) BSA/ 0.1% (vol/vol) Triton-X100 before being incubated at 4 °C overnight with primary antibodies directed against integrin β4 (mouse monoclonal, Abcam) E-cadherin (mouse monoclonal, Abcam), ZO-1 (mouse monoclonal, Life technology), VE-cadherin (rabbit polyclonal, Abcam), mucin 2 (mouse monoclonal, Santa Cruz Biotechnology), mucin 5AC (mouse monoclonal, Thermo Fisher Scientific), lysozyme (rabbit polyclonal, Dako), chromogranin A (goat polyclonal, Santa Cruz Biotechnology), villin (monoclonal mouse, Abcam), NHE3 (rabbit polyclonal, Novus Biologicals), alpha 1 Sodium/Potassium ATPase (mouse monoclonal, Abcam) and Ki67 (rabbit polyclonal, Abcam). Appropriate Alexa Fluor secondary antibodies were flowed into the two channels of the chip and incubated in the dark at 4 °C overnight. Images were acquired with an inverted laser-scanning confocal microscope (Leica SP5 X MP DMI-6000).

SEM samples were fixed in 2.5% glutaraldehyde, treated with 1% osmium tetroxide in 0.1 M sodium cacodylate buffer, dehydrated in a graded series of ethanols and critical point dried (AutoSamdri-815, Tousimis Research Corp.). Samples were coated with a thin (10 nm) layer of Pt/Pd using a sputter coater (Leica Baltec MED-020, Leica, Wetzlar, Germany) prior to imaging using a SEM (Zeiss Supra 55 VP SEM, Carl Zeiss SMT Inc). Epithelial morphology was evaluated using DIC microscopy (Zeiss Axio Observer Z1 2, AXIO2) or laser scanning confocal immunofluorescence microscopy (Leica SP5 X MP DMI-6000 and Zeiss TIRF/LSM 710). After sectioning the chips using a sharp surgical blade, images of the cross sections were acquired with a confocal immunofluorescence microscope and high-resolution images were obtained applying deconvolution (Huygens) followed by a 2D projection processing. High-resolution top-view images of Intestine Chips and Organoids were used to quantify villi-like structure morphology using the Fiji image processing package of ImageJ^47–49^.

### RNA isolation, reverse transcription and qRT-PCR

Epithelial RNA recovered from the Intestine Chip was extracted using RNeasy Mini Kit followed by cDNA synthesis with SuperScript VILO cDNA Synthesis Kit (Thermo Fisher Scientific). RT-PCR was performed using TaqMan Fast Advanced Master Mix (Applied Biosystems), TaqMan gene expression assays (Thermo Fisher Scientific; Hs00357579_g1 for intestinal-type alkaline phosphatase (ALPI), Hs00356112_m1 for sucrase isomaltase (SI), Hs00873651_g1 for mucin 5AC (MUC5AC), Hs00894025_m1 for mucin 2 (MUC2), Hs00900375_m1 for chromogranin A (CHGA), Hs00300531_m1 for synaptophysin (SYP), Hs00426232_m1 for lysozyme (LYZ), Hs00969422_m1 for leucine-rich-repeat-containing G-protein-coupled receptor 5 (LGR5), Hs00409825_g1 for Bmi1 (BMI1), Hs02758991_g1 for glyceraldehyde-3-phosphate dehydrogenase (GAPDH)), and run on a QuantStudio 7 Flex Real-Time PCR System (Thermo Fisher Scientific). Results were normalized relative to GAPDH expression.

### Gene microarray studies and differential expression analysis

Total RNA samples (100 ng) were processed using the GeneChip WT PLUS Reagent Kit and hybridized to Affymetrix Human Clariom D arrays. Current and past samples of Intestine Chips, Caco-2 Gut Chips and Caco-2 Transwells, as well as human intestinal tissues from GEO, were pre-processed with SCAN (SCAN.UPC package)^50,51^. Resulting expression values were then quantile-normalized together, and differential analysis was performed with limma package for each comparison pair^52^; gene expression values were averaged for each condition. Template matching was used to extract genes that are differentially expressed between these conditions^53,54^. Gene lists for the selected GO terms were collected from the Gene Ontology Consortium (http://geneontology.org/). For each GO term, no more than 18 genes were selected to determine similarity between Intestine Chip and duodenum as well as differences compared to the other samples based on their z-score and template matching score [PMID 11597334], and a curated heatmap for these selected genes grouped by GO terms was generated by clustering the conditions according to the averaged gene expression values using Canberra distance and complete linkage. Gene expression data for primary human small intestine (duodenum, jejunum, and ileum), Caco-2 Gut Chip, and Caco-2 Transwell were obtained from the National Center for Biotechnology Information (NCBI) Gene Expression Omnibus (GEO) database^55^ (accession no. GSE65790^21^). Transcriptome profiles of duodenal organoids and primary Intestine Chip performed in this study were deposited to the NCBI GEO database (accession no. GSE109471). Global gene expression profiles were visually represented using self-organizing maps generated using the Gene Expression Dynamics Inspector (GEDI) program^56^. In GEDI, each pixel tile within a mosaic represents a minicluster of genes that have highly similar expression patterns across all analyzed samples. The same genes are forced to the same mosaic position for all GEDI maps, hence allowing direct comparison of transcriptomes based on the overall mosaic pattern. The color of tiles indicates the centroid value of gene expression level for each minicluster.

### Statistical analysis

Either a Student’s t-test or 2-way ANOVA was performed to determine statistical significance, as indicated in the figure legends (error bars indicate standard error of the mean (SEM); *P* values < 0.05 were considered to be significant). Comparison of correlations was performed using R package cocor [PMID25835001].

## Electronic supplementary material

Supplementary Information
