# Supplementary material for: Development of a primary human Small Intestine-on-a-Chip using biopsy-derived organoids
Source: Sci Rep. 2018 Feb 13;8:2871. doi: 10.1038/s41598-018-21201-7 (PMC5811607; doi:10.1038/s41598-018-21201-7)
Supplement: Supplementary file 1 — Supplementary Information [file 41598_2018_21201_MOESM1_ESM.pdf]

SUPPLEMENTAL INFORMATION

Development of a primary human Small Intestine-on-a-Chip using biopsy-derived organoids

Magdalena Kasendra[1,+,†], Alessio Tovaglieri[1,2,+], Alexandra Sontheimer-Phelps[1,3], Sasan Jalili-Firoozinezhad[1,4], Amir Bein[1], Angeliki Chalkiadaki[1], William Scholl[1], Cheng Zhang[5], Hannah Rickner[6], Camilla A. Richmond[7,8], Hu Li[5], David T. Breault[6,8,9], and Donald E. Ingber[1,10,11*]

[1]Wyss Institute for Biologically Inspired Engineering at Harvard University, Boston, MA, USA.
[2]Graduate program, Department of Health Sciences and Technology, ETH Zurich, Zurich, Switzerland.
[3]Graduate program, Faculty of Biology, University of Freiburg, Freiburg, Germany
[4]Department of Bioengineering and iBB - Institute for Bioengineering and Biosciences, Instituto Superior Técnico, Universidade de Lisboa, Lisboa, Portugal
[5]Department of Molecular Pharmacology and Experimental Therapeutics, Mayo Clinic College of Medicine, Rochester, MN 55905, USA
[6]Division of Endocrinology, Boston Children's Hospital, Boston, MA, USA.
[7]Division of Gastroenterology, Boston Children's Hospital, Boston, MA 02115 USA
[8]Department of Pediatrics, Harvard Medical School, Boston, MA, 02115 USA
[9]Harvard Stem Cell Institute, Cambridge, Boston, MA, USA.
[10]Harvard John A. Paulson School of Engineering and Applied Sciences, Harvard University, Cambridge, MA, USA.
[11]Vascular Biology Program and Department Surgery, Boston Children's Hospital and Harvard Medical School, Boston, MA, USA.
†Present address: Emulate Inc., 27 Drydock Avenue, Boston, MA 02210, USA
+these authors contributed equally to this work

Address all correspondence to: Donald E. Ingber, M.D.,Ph.D/, Wyss Institute at Harvard University, CLSB5, 3 Blackfan Circle, Boston, MA 02115 (em: don.ingber@wyss.harvard.edu; tel: 617-432-7044; fax: 617-432-7828)

SUPPLEMENTARY FIGURE LEGENDS

Supplementary Figure S1. Time course and extent of villus differentiation in the Intestine Chip. Representative phase contrast images of duodenal organoid-derived epithelial cells cultured on chip under continuous flow and peristalsis-like motions. **(a)** Comparison of the same field of view (center of the channel) imaged at 1, 4, 8 and 12 days of culture demonstrates that the villi formed *de novo* within these cultures. **(b)** Views of three different regions beginning (top), middle (center) and end (bottom) of the culture channel confirming that villi-like structures form at high density along the entire length of the channel.

Supplementary Figure S2. Genome-wide hierarchical clustering of the Intestine Chip versus other intestinal culture models. Hierarchical clustering analysis of genome-wide transcriptome profiles of Intestine Chip, Organoid, Caco-2 Gut Chip or Caco-2 Transwell cultured in static condition compared with normal human small intestinal tissues (Duodenum, Jejunum, and Ileum; microarray data from the published GEO database). The dendrogram was generated based on the averages calculated across all replicates, and all branches in the cluster have the approximately unbiased (AU) P value larger than 95. The y axis next to the dendrogram represents the metric for maximum distance between samples. Corresponding pseudocolored GEDI maps analyzing profiles of 650 metagenes between samples described above.

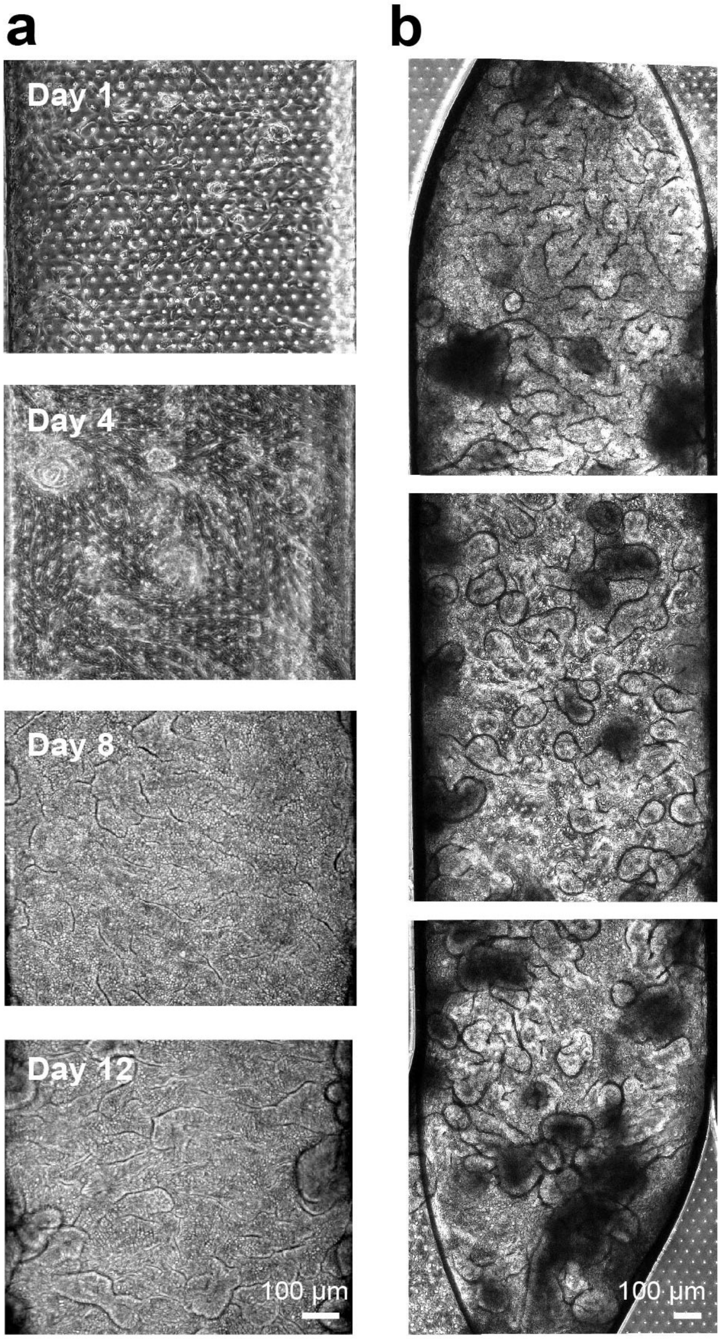

Supplementary Figure S1

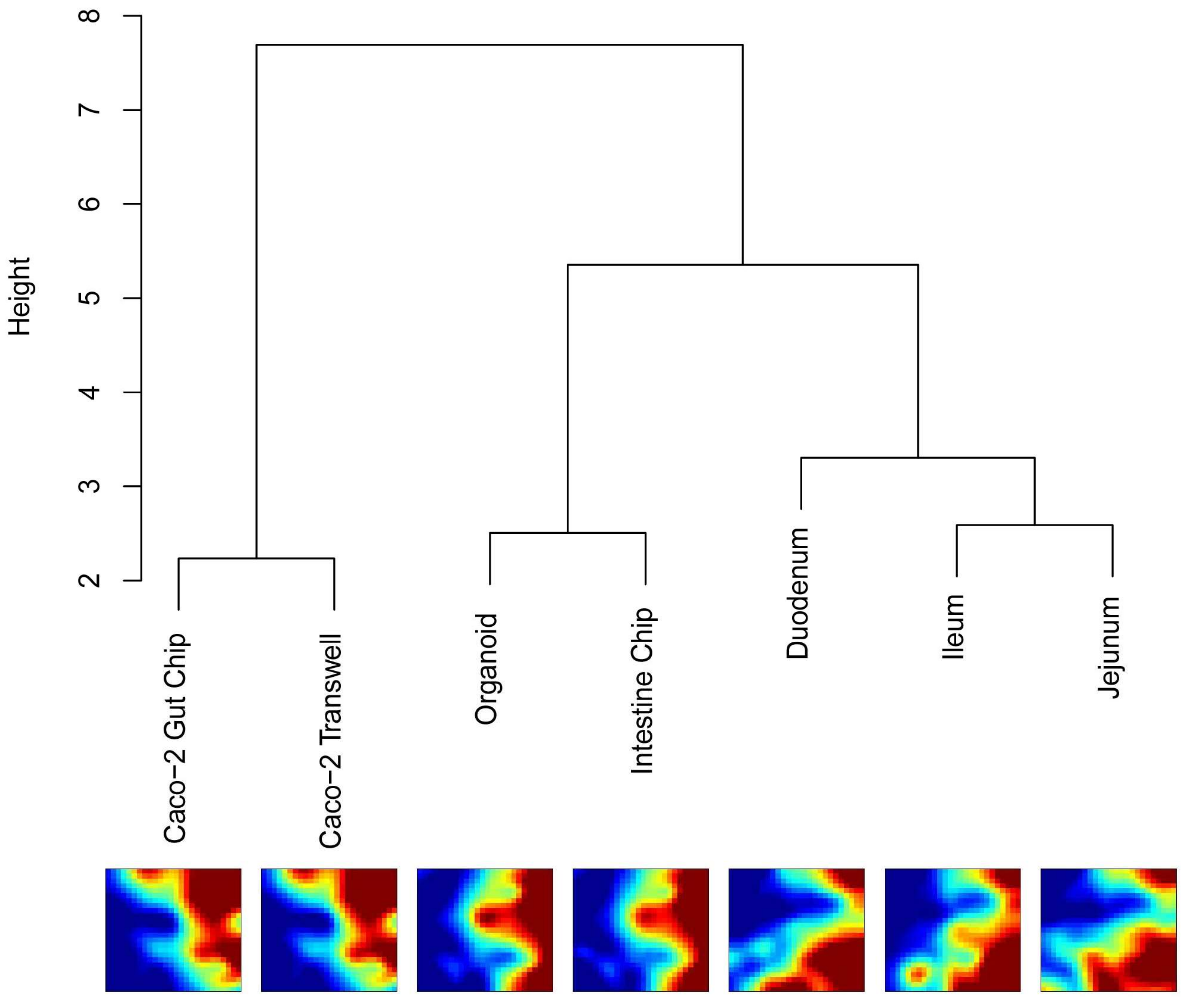

Supplementary Figure S2

SUPPLEMENTARY TABLE

Supplementary Table S1. List of genes used to generate the heatmap in Fig. 6.

GENE	Full Name	GO Term
ADAMTS13	ADAM metallopeptidase with thrombospondin type 1 motif 13	GO0006952_defense_response
NTHL1	nth-like DNA glycosylase 1	GO0006952_defense_response
HMGB1	high mobility group box 1	GO0006952_defense_response
AGER	advanced glycosylation end product-specific receptor	GO0006952_defense_response
IGLL1	immunoglobulin lambda like polypeptide 1	GO0006952_defense_response
PLA2G1B	phospholipase A2 group IB	GO0006952_defense_response
IGFBP4	insulin like growth factor binding protein 4	GO0006952_defense_response
CCL2	C-C motif chemokine ligand 2	GO0006952_defense_response
ELMO1	engulfment and cell motility 1	GO0006952_defense_response
CTSL	cathepsin L	GO0006952_defense_response
S1PR3	sphingosine-1-phosphate receptor 3	GO0006952_defense_response
PM20D1	peptidase M20 domain containing 1	GO0006952_defense_response
CTSB	cathepsin B	GO0006952_defense_response
PTX3	pentraxin 3	GO0006952_defense_response
SERPINE1	serpin family E member 1	GO0006952_defense_response
BMP6	bone morphogenetic protein 6	GO0006952_defense_response
JAM3	junctional adhesion molecule 3	GO0006952_defense_response
ICAM2	intercellular adhesion molecule 2	GO0006952_defense_response
SLC22A5	solute carrier family 22 member 5	GO0015893_drug_transport
ATP8B1	ATPase phospholipid transporting 8B1	GO0015893_drug_transport
MFSD10	major facilitator superfamily domain containing 10	GO0015893_drug_transport
SLC22A1	solute carrier family 22 member 1	GO0015893_drug_transport
ABCB4	ATP binding cassette subfamily B member 4	GO0015893_drug_transport
SLC47A1	solute carrier family 47 member 1	GO0015893_drug_transport
SLC22A2	solute carrier family 22 member 2	GO0015893_drug_transport
SLC38A7	solute carrier family 38 member 7	GO0015893_drug_transport
SLC38A1	solute carrier family 38 member 1	GO0015893_drug_transport
SLC19A3	solute carrier family 19 member 3	GO0015893_drug_transport
SLC19A2	solute carrier family 19 member 2	GO0015893_drug_transport
SLC38A3	solute carrier family 38 member 3	GO0015893_drug_transport
MUC6	mucin 6, oligomeric mucus/gel-forming	GO0022600_digestive_system_process
SLC22A5	solute carrier family 22 member 5	GO0022600_digestive_system_process
APOA4	apolipoprotein A-IV	GO0022600_digestive_system_process
CD36	CD36 molecule	GO0022600_digestive_system_process
NR1H3	nuclear receptor subfamily 1 group H member 3	GO0022600_digestive_system_process

AKR1C1	aldo-keto reductase family 1, member C1	GO0022600_digestive_system_process
TAC4	tachykinin 4 (hemokinin)	GO0022600_digestive_system_process
WNK4	WNK lysine deficient protein kinase 4	GO0022600_digestive_system_process
MUC4	mucin 4, cell surface associated	GO0022600_digestive_system_process
ACO1	aconitase 1	GO0022600_digestive_system_process
OPRL1	opioid related nociceptin receptor 1	GO0022600_digestive_system_process
PAWR	pro-apoptotic WT1 regulator	GO0022600_digestive_system_process
NKX3-1	NK3 homeobox 1	GO0050678_regulation_of_epithelial_cell_proliferation
AGER	advanced glycosylation end product-specific receptor	GO0050678_regulation_of_epithelial_cell_proliferation
MAP2K5	mitogen-activated protein kinase kinase 5	GO0050678_regulation_of_epithelial_cell_proliferation
ITGB3	integrin subunit beta 3	GO0050678_regulation_of_epithelial_cell_proliferation
SPARC	secreted protein acidic and cysteine rich	GO0050678_regulation_of_epithelial_cell_proliferation
BMP6	bone morphogenetic protein 6	GO0050678_regulation_of_epithelial_cell_proliferation
CD109	CD109 molecule	GO0050678_regulation_of_epithelial_cell_proliferation
ACVRL1	activin A receptor like type 1	GO0050678_regulation_of_epithelial_cell_proliferation
ENG	endoglin	GO0050678_regulation_of_epithelial_cell_proliferation
KDR	kinase insert domain receptor	GO0050678_regulation_of_epithelial_cell_proliferation
CAV1	caveolin 1	GO0050678_regulation_of_epithelial_cell_proliferation
FLT4	fms related tyrosine kinase 4	GO0050678_regulation_of_epithelial_cell_proliferation
EGFL7	EGF like domain multiple 7	GO0050678_regulation_of_epithelial_cell_proliferation
CCL2	C-C motif chemokine ligand 2	GO0050678_regulation_of_epithelial_cell_proliferation
PLAU	plasminogen activator, urokinase	GO0050678_regulation_of_epithelial_cell_proliferation
MYDGF	myeloid-derived growth factor	GO0050678_regulation_of_epithelial_cell_proliferation
HTR2B	5-hydroxytryptamine receptor 2B	GO0050678_regulation_of_epithelial_cell_proliferation
A4GNT	alpha-1,4-N-acetylglucosaminyltransferase	GO0050678_regulation_of_epithelial_cell_proliferation
C2	complement component 2	GO0007584_response_to_nutrient
TTPA	tocopherol (alpha) transfer protein	GO0007584_response_to_nutrient
LIPG	lipase G, endothelial type	GO0007584_response_to_nutrient
PAWR	pro-apoptotic WT1 regulator	GO0007584_response_to_nutrient
OGT	O-linked N-acetylglucosamine (GlcNAc) transferase	GO0007584_response_to_nutrient
VLDLR	very low density lipoprotein receptor	GO0007584_response_to_nutrient
SPARC	secreted protein acidic and cysteine rich	GO0007584_response_to_nutrient
CAV1	caveolin 1	GO0007584_response_to_nutrient
TXN2	thioredoxin 2	GO0007584_response_to_nutrient
ALDH1A2	aldehyde dehydrogenase 1 family member A2	GO0007584_response_to_nutrient
MOG	myelin oligodendrocyte glycoprotein	GO0007584_response_to_nutrient
OXCT1	3-oxoacid CoA-transferase 1	GO0007584_response_to_nutrient
PIM1	Pim-1 proto-oncogene, serine/threonine kinase	GO0007584_response_to_nutrient
OTC	ornithine carbamoyltransferase	GO0007584_response_to_nutrient
